# Highly specific inhibition of leukaemia virus membrane fusion by interaction of peptide antagonists with a conserved region of the coiled coil of envelope

**DOI:** 10.1186/1742-4690-5-70

**Published:** 2008-08-04

**Authors:** Daniel Lamb, Alexander W Schüttelkopf, Daan MF van Aalten, David W Brighty

**Affiliations:** 1 The Biomedical Research Centre, College of Medicine, Ninewells Hospital, The University, Dundee, DD1 9SY, Scotland, UK; 2 The Division of Biological Chemistry and Drug Discovery, College of Life Sciences, University of Dundee, Dow Street, Dundee, DD1 5EH, Scotland, UK

## Abstract

**Background:**

Human T-cell leukaemia virus (HTLV-1) and bovine leukaemia virus (BLV) entry into cells is mediated by envelope glycoprotein catalyzed membrane fusion and is achieved by folding of the transmembrane glycoprotein (TM) from a rod-like pre-hairpin intermediate to a trimer-of-hairpins. For HTLV-1 and for several virus groups this process is sensitive to inhibition by peptides that mimic the C-terminal α-helical region of the trimer-of-hairpins.

**Results:**

We now show that amino acids that are conserved between BLV and HTLV-1 TM tend to map to the hydrophobic groove of the central triple-stranded coiled coil and to the leash and C-terminal α-helical region (LHR) of the trimer-of-hairpins. Remarkably, despite this conservation, BLV envelope was profoundly resistant to inhibition by HTLV-1-derived LHR-mimetics. Conversely, a BLV LHR-mimetic peptide antagonized BLV envelope-mediated membrane fusion but failed to inhibit HTLV-1-induced fusion. Notably, conserved leucine residues are critical to the inhibitory activity of the BLV LHR-based peptides. Homology modeling indicated that hydrophobic residues in the BLV LHR likely make direct contact with a pocket at the membrane-proximal end of the core coiled-coil and disruption of these interactions severely impaired the activity of the BLV inhibitor. Finally, the structural predictions assisted the design of a more potent antagonist of BLV membrane fusion.

**Conclusion:**

A conserved region of the HTLV-1 and BLV coiled coil is a target for peptide inhibitors of envelope-mediated membrane fusion and HTLV-1 entry. Nevertheless, the LHR-based inhibitors are highly specific to the virus from which the peptide was derived. We provide a model structure for the BLV LHR and coiled coil, which will facilitate comparative analysis of leukaemia virus TM function and may provide information of value in the development of improved, therapeutically relevant, antagonists of HTLV-1 entry into cells.

## Background

Bovine Leukemia Virus (BLV) and Human T-Cell Leukemia Virus Type-1 (HTLV-1) are closely related deltaretroviruses that cause aggressive lymphoproliferative disorders in a small percentage of infected individuals [1-3]. In order to efficiently enter cells, both viruses are dependent on a fusion event between viral and cell membranes. As with other retroviruses, fusion is catalyzed by the virally encoded Env complex, which is synthesized as a polyprotein precursor and is subsequently cleaved to yield the surface glycoprotein (SU) and transmembrane glycoprotein (TM) subunits. On the surface of the virus or infected cell, Env is displayed as a trimer, with three SU subunits linked by disulphide bonds to a spike of three TM subunits.

The amino-acid sequences of the HTLV-1 and BLV envelope glycoproteins are strikingly similar [4] and, in common with other oncoretroviruses, share a characteristic modular structure [4-8]. A receptor-binding domain is located at the amino-terminal end of SU and is connected to a C-terminal domain by a proline-rich linker [4,6,9]. The C-terminal domain includes a conserved CXCC sequence and is required for interactions with TM [10-12]. The modular nature of envelope extends into TM, and it is here that the homology between retroviruses and phylogenetically diverse viral isolates is most apparent. The functional regions of TM include a hydrophobic fusion peptide linked to an isoleucine/leucine heptad repeat, a membrane spanning segment and a cytoplasmic tail of variable length. These conserved modules identify retroviral TM proteins as members of a diverse family of virally expressed class 1 membrane fusion proteins.

Accumulating evidence advocates a conserved mechanism of retroviral envelope-mediated membrane fusion [13-15]. SU binds to the cellular receptor, which is accompanied by isomerisation of the disulphide linkages between SU and TM [11,12], and triggers a conformational change in TM. The N-terminal hydrophobic fusion peptide of TM is then inserted into the target cell membrane, while the C-terminus remains anchored in the viral or host cell membrane. This transient rod-like conformation, referred to as a "pre-hairpin" intermediate, is stabilized by the assembly of a trimeric coiled coil composed of one alpha helix from each of the three adjacent TM monomers. A more C-terminal region of the TM ecto-domain, which in HTLV-1 includes an extended non-helical leash and short α-helix [16], then folds onto the coiled coil to generate a six-helix bundle or trimer-of-hairpins [16-19]. These dramatic conformational changes draw the opposing membranes together, destabilise the lipid bilayers, promote lipid mixing and culminate in membrane fusion [13,14].

Despite the sequence homology and conserved modular structure, there are notable differences in primary sequence, size, and function of the HTLV-1 and BLV envelope proteins. It is likely that these differences contribute in a substantial way to the species-specificity, and the distinctive patterns of tissue tropism and pathogenesis that are observed for these viruses [2,3]. Consequently, comparative analysis of the envelope glycoproteins will provide significant insight into the determinants of species- and tissue-specific tropism, the strategies for immune modulation, and the mechanisms of membrane fusion that are adopted by these viruses. Information derived from such studies will aid the development of effective vaccines and small-molecule inhibitors of viral entry and cell-to-cell viral transfer.

Significantly, our laboratory [20-22], and others [23], have demonstrated that synthetic peptides that mimic the C-terminal non-helical leash and α-helical region (LHR) of HTLV-1 TM are inhibitory to envelope-mediated membrane fusion. Prototypic α-helical TM-mimetic inhibitory peptides have also been characterized for a number of highly divergent enveloped viruses, including HIV and paramyxoviruses [24-27]. The HTLV-derived peptide binds to the coiled coil of TM and, in a *trans*-dominant negative manner, blocks resolution of the pre-hairpin intermediate to the trimer-of-hairpins, thus impairing the fusogenic activity of TM. The potency of these inhibitors makes them attractive leads for antiviral therapeutics.

Although the HTLV-1 peptide inhibitor also blocks viral entry of the divergent HTLV-2 it is inactive against a variety of heterologous viral envelope proteins [20,23]. However, the molecular features that determine the target specificity, activity, and potency of these peptide inhibitors is only beginning to be understood [20-22]. In this study, we examine the target specificity and activity of peptide inhibitors derived from the conserved C-terminal leash and α-helical region (LHR) of the HTLV-1 and BLV trans-membrane glycoproteins. We demonstrate that a synthetic peptide that mimics the BLV LHR is a potent antagonist of BLV envelope-mediated membrane fusion. Surprisingly, despite the high level of identity between the HTLV-1 and BLV derived peptides, the inhibitory activity of the peptides is limited exclusively to the virus from which they were derived. While the peptides display remarkable target specificity, the activity of each peptide is nevertheless dependent upon the interaction of conserved amino acid side chains with their respective targets. An amino acid substitution analysis reveals that several conserved residues within the BLV LHR play a critical role in determining peptide potency and identifies a single amino acid substitution within the BLV peptide that yields a more potent inhibitor. Finally, based on homology with HTLV-1 TM, the inhibition data and amino acid substitution analysis support a model for the BLV trimer-of-hairpins.

## Materials and methods

### Cells

HeLa and BLV-FLK (a kind gift of Dr Arsène Burny and Dr Luc Willems; Universitaire des Sciences Agronomiques de Gembloux, Belgium) cells were maintained in Dulbecco's modified Eagle medium supplemented with 10% fetal bovine serum (FBS).

### Plasmids

The Plasmid HTE-1 [28] and pRSV-Rev [29] have been described. The plasmid pCMV-BLV*env*-RRE was constructed by replacing a fragment of the HIV-1 envelope open reading frame in pCMVgp160ΔSA [30] with a genomic fragment spanning the entire BLV envelope. In brief, pCMVgp160ΔSA was digested with EcoR I, which cuts the recipient vector after the CMV early promoter but prior to the initiating ATG of the HIV-1 *env *sequences. The vector was subsequently digested with BglII, which removes the HIV-1 SU region but retains the HIV RRE. A fragment encompassing the entire BLV envelope open reading frame between a 5' Xho I site and a 3' BamH I site (nucleotides 4347–6997 of NC_001414) was ligated into the vector backbone using an EcoR I-Xho I linker. The resulting plasmid encodes BLV *env *including the natural BLV *env *stop codon placed upstream of the HIV RRE; the transcription unit is terminated by the SV40 poly A site and is expressed from the CMV early promoter.

### Peptides

Peptides (Table 1) were synthesized using standard solid-phase Fmoc chemistry and unless stated otherwise have acetylated N-termini and amidated C-termini. The peptides were purified by reverse-phase high-pressure liquid chromatography and verified for purity by MALDI-TOF mass spectrometry. All peptides were dissolved in dimethyl sulfoxide (DMSO), the concentration of peptide stock solutions was confirmed where possible by absorbance at 280 nm in 6 M guanidine hydrochloride and peptides were used at the final concentrations indicated. For the peptide P^BLV^-ΔN, peptide concentration was estimated by Bradford assay at 5 two-fold serial dilutions from a stock solution using the P^BLV^-ΔC peptide in concentrations verified by absorbance at 280 nm in 6 M guanidine hydrochloride to plot a standard curve. The HTLV-1-derived peptides are based on the sequence of HTLV-1 strain CR and conform closely to the consensus sequence for HTLV-1 and HTLV-2 strains, the BLV peptides conform to the consensus sequence for most BLV isolates.

**Table 1:** Peptides used in this study.

Peptide	Amino Acid Position	Sequence	MW	Maximum Solubility (μM)*
P^cr^-400	gp21 400–429	CCFLNITNSHVSILQERPPLENRVLTGWGL	3,411	> 90.00
P^cr^-400 L/A	gp21 400–429	---A---------A-----A----A----A	3,200	45.00
P^BLV^-391	gp30 391–419	CCFLRIQNDSIIRLGDLQPLSQRVSTDWQ	3,447	> 90.00
P^BLV^-ΔN	gp30 400–419	S-------------------	2,312	> 90.00
P^BLV^-ΔC	gp30 391–410	-------------------L	2,317	> 90.00
P^BLV^-L/A	gp30 391–419	---A---------A--A--A---------	3,236	45.00
P^BLV^-L404/410A	gp30 391–419	-------------A-----A---------	3,321	> 90.00
P^BLV^-ΔCCF	gp30 394–419	L-------------------------	3,052	11.25
P^BLV^-R403A	gp30 391–419	------------A----------------	3,321	22.50
C34	gp41 627–661	GWMEWDREINNYTSLLIHSLIEESQNQQEKNEQELL	4,418	> 90.00

* Maximum solubility in aqueous solution determined by laser nephelometry.

### Peptide biotinylation

Peptides to be biotinylated were reduced using immobilized Tris [2-carboxyethyl] phosphine (TCEP) reducing agent (Pierce), and subsequent biotinylation was carried out with EZ-Link^® ^Iodoacetyl-PEO_2_-Biotin (Pierce), in both cases according to the manufacturer's protocols. The biotinylation reaction was quenched with cysteine. The biotinylated peptide was incubated for 30 mins at room temperature with either streptavidin-agarose (Gibco-BRL) or amylose-agarose (New England Biolabs) in a spin-column. Unbound peptide was recovered by centrifugation, the flow-through was re-applied to the column, and the incubation and centrifugation was repeated. The flow-through from the second centrifugation was used in syncytium interference assays; the peptide concentration of the amylose-agarose flow through was established by UV spectrometry as described above, and added to tissue culture medium to produce the final assay concentrations as indicated. In the case of the flow-through from the streptavidin-agarose column, volumes equivalent to those used with the amylose-agarose flow-through were added to the wells.

### Determination of relative peptide solubility

A two-fold serial dilution of peptide in DMSO was performed, and added in duplicate to 96-well microplates. Filtered PBS was added to give a total volume of 200 μl and a final DMSO concentration of 1.5 % in all wells. The plates were incubated at room temperature for 1 hr and the relative solubility of peptides was established by measuring forward scattered light using a NEPHELOstar laser-based microplate nephelometer (BMG LABTECH). Wells containing PBS and 1.5 % DMSO only were used as blanks. Data analysis was carried out using ActivityBase, and peptides giving readings up to and including 3-fold higher than the average reading for the DMSO control were considered to be in solution at the concentrations specified.

### Syncytium Interference Assays

Syncytium interference assays were performed by standard methods [20,31]. Briefly, HeLa cells for use as effector cells were transfected with the envelope expression vector pHTE-1 or with equal amounts of pCMV-BLV*env*-RRE and pRSV-Rev using the Genejuice™ transfection reagent (Novagen) in accordance with the manufacturer's instructions. 24 h later, 3 × 10^5 ^effector cells were added to 7 × 10^5 ^untransfected HeLa target cells in six-well dishes (Nunc). Where appropriate, the co-culture was incubated in the presence of peptides at the concentrations specified. To assess the ability of the peptides to inhibit fusion induced by virally expressed BLV envelope, 2 × 10^5 ^BLV-infected FLK cells were used as effectors and added to 8 × 10^5 ^uninfected HeLa cells. After incubation at 37°C for 16 h, cells were washed twice with PBS and fixed in PBS + 3% paraformaldehyde. Assays were performed in triplicate and the number of syncytia (defined as multinucleated cells with 4 or more nuclei) from 10 low-power fields (LPF) per replicate was scored by light microscopy; some assays were stained using Giemsa. A syncytium formation value of 100% is defined as the number of syncytia formed in the absence of peptide but in the presence of 1.5% DMSO. The peptide concentration required to give 50% inhibition (IC_50_) of syncytium formation was calculated using GraphPad Prism 4.

## Results

### Amino acid residues conserved between the HTLV-1 and BLV TM ectodomains map to the interacting surfaces of the LHR and coiled-coil

**Figure 1:**
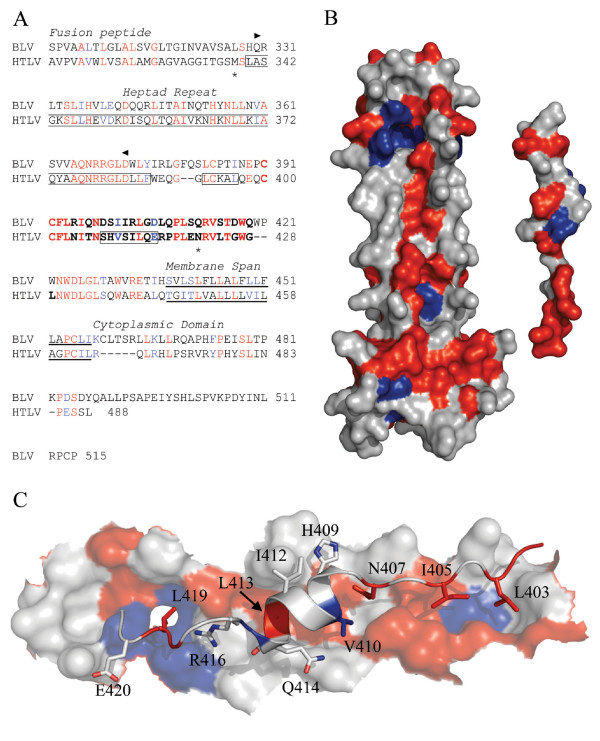
**Analysis of the conserved regions of BLV and HTLV-1 TM**. (A) Alignment of the BLV and HTLV-1 TM sequences, the predicted coiled coil of BLV TM is indicated between the arrow heads; the LHR is in bold; the helical regions of the HTLV-1 TM are boxed; the limits of the HTLV-1 crystal structure are marked by asters; and the membrane spanning region is underlined. (B) The HTLV-1 core coiled-coil and, on the right, the leash and α-helical region that is mimicked by the HTLV-1 inhibitory peptide (from PDB 1MG1). The face of the peptide that interacts with the coiled coil is shown. For the sequence alignment and structural renderings, residues identical between BLV and HTLV-1 are shown in red, conservative substitutions are blue, and non-conserved are rendered white. Amino acid coordinates refer to the full-length envelope precursor. (C) Detail of the predicted interaction of the HTLV-1 LHR-mimetic peptide (ribbon structure) with the surface of the coiled coil (space filling form) based on the structure of Kobe *et al*. [16]; shading as above.

Although there are considerable differences in the amino acid sequence of class-1 fusion proteins from diverse viral groups there is exceptional conservation of secondary and tertiary structure. To compare the class-1 fusion proteins from the related retroviruses BLV and HTLV-1, the predicted coiled-coil regions of the BLV TM were identified using the program LearnCoil-VMF [32] and the BLV and HTLV-1 amino acid sequences were aligned using Clustal-W [33] (Figure 1A). The alignment revealed that for the TM 33% of the residues are identical and a further 10% are conservative substitutions. The homology is particularly evident in the predicted coiled-coil region incorporating the heptad repeat and in the LHR of the TM ectodomain (Figure 1A), the LHR lies distal to a CX_6_CC motif common to oncoretroviral fusion proteins. The crystal structure of the HTLV-1 six-helix-bundle has been solved and the structure spans these regions of homology [16].

Using the crystal structure of the HTLV-1 TM as a template, we mapped on the coiled coil and LHR the location of amino acid residues that are conserved between the ectodomain of HTLV-1 and BLV TM (Figure 1B). Using this approach, we observed that for the core coiled-coil the majority of conserved residues map along the grooves formed by the interface of each pair of interacting N helices. Importantly, these grooves act as docking sites for the LHR as TM folds from the pre-hairpin intermediate to the trimer-of-hairpins. Moreover, many of the conserved amino acids of the LHR are located on the face of the LHR that interacts with the grooves on the coiled coil. By examining the location of substituted residues on the HTLV-1 TM it becomes clear that where there are amino acid substitutions on the BLV LHR there are complimentary or accommodating amino acid changes within the hydrophobic grooves of the core coiled coil (Figure 1C). For example, leucines 413 and 419 in the HTLV-1 LHR are conserved in BLV, and these leucines interact with eight coiled coil residues of which seven are identical in BLV and one is a conservative substitution (Figure 1C). In contrast, HTLV-1 LHR residues H409 and R416 interact with the side chains of six residues of the coiled coil, but H409 and R416 are not conserved in BLV and of the six interacting coiled coil residues four have diverged and only one residue is semi-conserved (Figure 1C). Overall, the analysis indicates that the majority of the conserved residues occupy positions that form the interacting surfaces of the trimer-of-hairpins. In agreement with these observations, those residues that do not involve the interacting surfaces of the TM are invariably solvent exposed on the trimer-of-hairpins and are subject to the highest degree of variation between the two viruses.

A synthetic peptide, P^cr^-400, which mimics the LHR of the HTLV-1 TM is a potent inhibitor of envelope-catalysed membrane fusion [20]. This peptide interacts directly and specifically with a recombinant coiled coil derived from HTLV-1 TM and substitution of critical amino acid residues within the peptide disrupts coiled coil binding and impairs the biological activity of the peptide [20-22]. These findings are consistent with the view that the peptide blocks membrane fusion by binding to the coiled coil of fusion-active envelope. As illustrated above, there are remarkable similarities in the interacting surfaces of the coiled coil and LHR between HTLV-1 and BLV (Figure 1). Considering the noted differences, it was not clear if the HTLV-1-derived synthetic peptide could inhibit membrane fusion mediated by BLV envelope. The HTLV-1 peptide inhibits viral entry by the divergent HTLV-2 but does not inhibit membrane fusion catalysed by a number of heterologous viral envelopes including HIV-1, feline immunodeficiency virus and vesicular stomatitis virus G protein [20,23] (our unpublished results). Moreover, the HTLV-1 inhibitory peptide is unusual among C helix-based fusion inhibitors in that it includes both α-helical and extended non-helical peptide segments. It was therefore uncertain if peptides based on the LHR of BLV would, like the HTLV-mimetic peptide, display anti-fusogenic activity. We therefore compared the fusogenic activity of HTLV-1 and BLV envelope and examined the sensitivity of BLV envelope to inhibition by peptide inhibitors.

### A robust BLV Env-mediated membrane fusion assay

**Figure 2:**
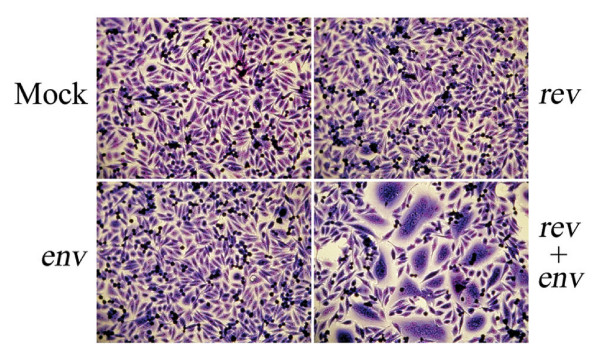
**BLV Env-induced syncytia**. Mock transfected HeLa cells (Mock) or HeLa cells transfected with pRSV-Rev alone (*rev*), pCMV-BLVenv-RRE alone (*env*), or both pRSV-Rev and pCMV-BLVenv-RRE (*rev *+ *env*) were co-cultured with target untransfected HeLa cells. Cells were stained with Giemsa and typical syncytia profiles are shown.

Preliminary experiments with a variety of BLV envelope expression constructs produced only low levels of BLV envelope expression and little fusogenic activity in syncytium formation assays (data not shown); this may, in part, be due to the nuclear retention of the envelope transcripts as observed for HIV-1 and HTLV-1. Therefore, we developed an envelope expression vector whereby BLV *env *was inserted downstream of the strong cytomegalovirus (CMV) early promoter, and immediately upstream of the human immunodeficiency virus Rev-response element (RRE). The RRE forms a region of extensive secondary structure in the mRNA that is recognized by Rev and the resulting ribonucleoprotein complex is subsequently exported out of the nucleus. The BLV envelope expression construct was examined for envelope-induced membrane fusion in syncytium formation assays. Briefly, HeLa cells were either transfected with pCMV-BLV*env*-RRE or pRSV-Rev individually, or cotransfected with equal amounts of both vectors. These cells were then used as effector cells to induce syncytia when co-cultured with non-transfected cells. Neither vector induced syncytium formation when transfected alone, but cotransfection of effector cells with pCMV-BLV*env*-RRE and pRSV-Rev resulted in the widespread formation of large syncytia (Figure 2). Furthermore, BLV envelope expressed in this system produced levels of syncytia that were comparable to that of HTLV-1 envelope expressed from pHTE-1 and consequently this approach was used to express BLV envelope for these studies.

### Inhibition of envelope-mediated membrane fusion by LHR-mimetic peptides is limited to the parental virus

To compare the inhibitory properties and specificity of LHR-based synthetic peptides from HTLV-1 and BLV a peptide based on the LHR of BLV was generated. The synthetic peptide designated P^BLV^-391 includes residues Cys391 to Gln419 of BLV Env and spans a region that is equivalent to the HTLV-1 LHR-derived peptide P^cr^-400 (Table 1). To aid comparison with TM, we refer to the residues of each peptide using the co-ordinates for the full-length envelope precursor (thus for the BLV-derived peptide residue 1 is referred to as Cys391). The BLV and HTLV-1 peptides share 45 % identity (Figure 1A, B), but it should be noted that only a fragment of the HTLV-1 LHR that is mimicked by P^cr^-400 is resolved in the available HTLV-1 TM crystal structure (Table 1, Figure 1) [20].

**Figure 3:**
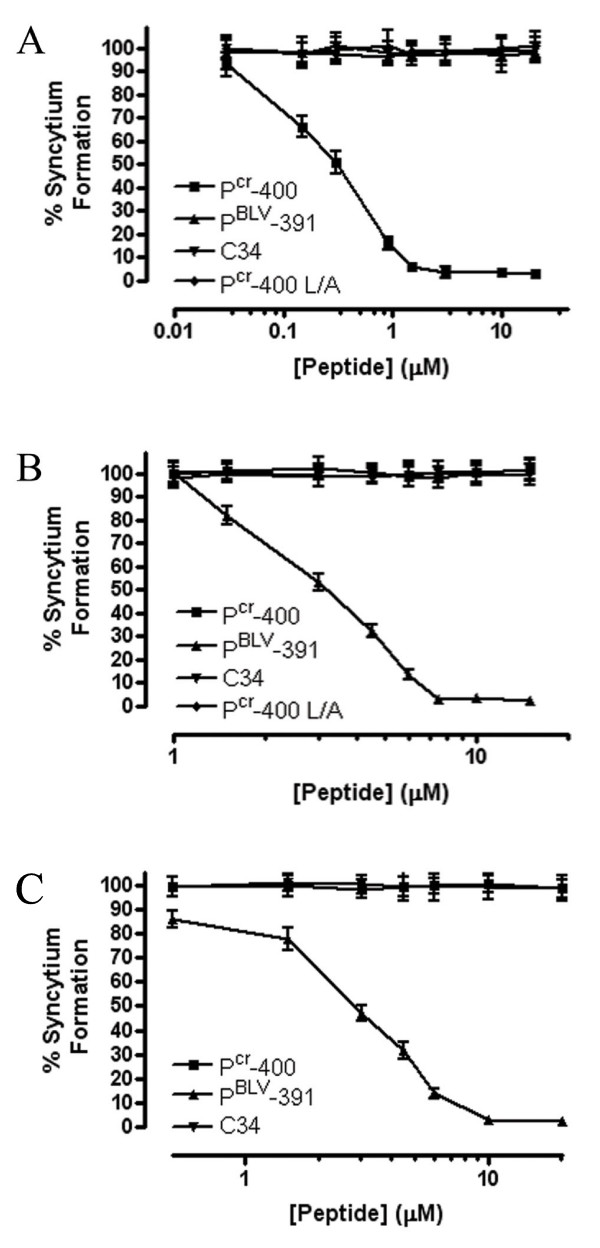
**The specificity of peptide inhibitors of Envelope-mediated membrane fusion is limited to the parental virus**. HeLa cells expressing HTLV-1 (A) or BLV (B) envelope were used as effector cells and co-cultured with untransfected HeLa cells. Cells were incubated in the presence of the peptides P^cr^-400, P^BLV^-391, P^cr^-400 L/A a non-functional derivative of P^cr^-400 [20], or the control HIV C helix mimetic peptide C34 [51]. (C) Syncytia formation between BLV infected FLK cells and non-infected HeLa cells. Syncytia were counted in 10 low-power light microscope fields. Data points show the mean ± SD of triplicate assays.

Both HTLV-1 and BLV envelope induced widespread syncytium formation in cultures incubated in the absence of peptide inhibitors or in the presence of inactive control peptides (Figure 3A, B). However, in keeping with previous studies [20-22], HTLV envelope-mediated syncytium formation was robustly blocked in a dose-dependent manner by P^cr^-400 with an IC_50 _of 0.28 ± 0.01 μM (Figure 3A). However, despite the marked conservation of amino acid sequence between the LHRs and coiled coils of HTLV-1 and BLV, P^cr^-400 failed to inhibit membrane fusion induced by BLV envelope even at concentrations up to 15 μM (Figure 3B) and above (data not shown). Also, like the inactive control peptides, the BLV LHR-mimetic peptide at concentrations up to 20 μM (Figure 3A) and above (data not shown) failed to inhibit membrane fusion induced by HTLV-1 envelope. By contrast, the peptide P^BLV^-391 specifically antagonized BLV envelope-mediated membrane fusion (Figure 3B) with a calculated IC_50 _of 3.49 ± 0.03 μM; control peptides including C34 and P^cr^-400 L/A did not interfere with BLV Env-induced membrane fusion (Figure 3B). In addition, P^BLV^-391 robustly antagonized membrane fusion induced by virally expressed envelope as shown by the inhibition of syncytium formation between chronically BLV infected FLK cells and target cells (Figure 3C); whereas, the HTLV-1 peptide inhibitor did not block BLV-induced membrane fusion. Thus, it appears that the inhibitory properties of the LHR-mimetic peptides are highly specific to the virus from which they were derived.

### The C- and N-terminal regions of P^BLV^-391 are necessary but not individually sufficient to block membrane fusion

**Figure 4:**
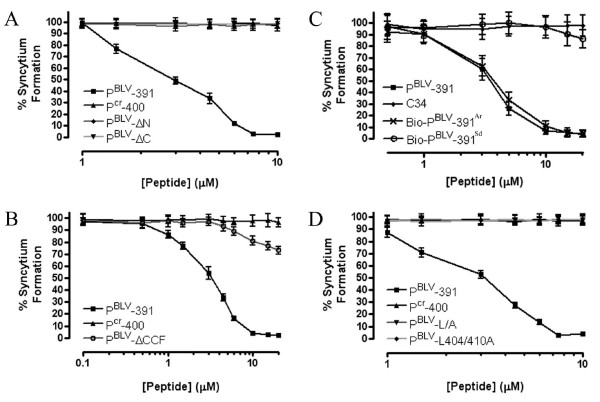
**Deletions or substitutions of specific amino acids in P^BLV^-391 have a detrimental effect on inhibitory activity**. Syncytium interference assays using BLV envelope-expressing HeLa cells as effectors. (A) The inhibitory properties of P^BLV^-391, P^BLV^-ΔN, P^BLV^-ΔC and the P^cr^-400 control were examined. (B) The activity of P^BLV^-391, the derivative P^BLV^-ΔCCF, and the control peptide P^cr^-400 were compared. (C) The activity of P^BLV^-391 was compared to Bio-P^BLV^-391^Ar ^a biotinylated peptide recovered from the flow-through of an amylose column (see methods), Bio-P^BLV^-391^Sd ^the same peptide depleted over a streptavidin column (volumes of column buffer equal to those required to give the specified concentrations of Bio-P^BLV^-391^Ar ^were used), and the control peptide C34. (D) The inhibitory properties of P^BLV^-391, P^BLV^-L/A, P^BLV^-L404/410A and the control P^cr^-400 were compared. Syncytia were counted in 10 low-power light microscope fields. Data points show the mean ± SD of triplicate assays.

Our group recently demonstrated that truncations at the N- or C-terminal end of P^cr^-400 abolished fusion-inhibitory function [29]. To test whether or not the N- and C-terminal leash regions are required for the activity of P^BLV^-391, we synthesized two peptides, P^BLV^-ΔN and P^BLV^-ΔC, which lack nine amino acid residues at the N-terminus or C-terminus respectively (Table 1). The peptides retain an eleven-residue overlap, and have solubility profiles comparable to the parental peptide P^BLV^-391 (Table 1). Unlike the parental peptide, the peptide derivatives P^BLV^-ΔN and P^BLV^-ΔC lacked detectable inhibitory activity in syncytium interference assays (Figure 4A). These data illustrate that amino acid residues contained within the regions Cys391 to Asp399, and Ser411 to Gln419, are critical to the activity of the mimetic peptide, and that both the amino-terminal and C-terminal regions are necessary but not sufficient for antagonism of membrane fusion. Importantly, the data also demonstrate that the central 11-residue region of the BLV peptide, equivalent to Ser400-Leu410 and homologous to the short C-terminal α-helix of the HTLV-1 trimer-of-hairpins is not sufficient for inhibition of syncytium formation.

Moreover, the BLV peptide was remarkably intolerant of even relatively small deletions. For example, a peptide, P^BLV^-ΔCCF, in which only 3 amino acids were deleted from the N-terminus exhibited dramatically reduced ability to inhibit membrane fusion (Figure 4B). The P^BLV^-ΔCCF peptide blocked syncytium formation by only 30% at 20 μM (Figure 4B), compared to > 95% for the parental peptide, and even at a concentration of 30 μM peptide P^BLV^-ΔCCF achieved only 40% inhibition (data not shown). These results can be explained only in part by the decrease in peptide solubility at concentrations above 11 μM that is associated with the loss of the three N-terminal amino acid residues (Table 1). At peptide concentrations below 11 μM, P^BLV^-ΔCCF is soluble under the conditions used in the syncytium interference assays and yet fails to inhibit membrane fusion (Figure 4B). It should be noted that disulphide formation between the peptide and envelope is not required for inhibitory activity, as reduction of P^BLV^-391 and subsequent modification of the cysteine residues with the sulfhydryl reactive agent Iodoacetyl-PEO_2_-Biotin failed to disrupt the inhibitory properties of the peptide (Figure 4C). Moreover, the activity of the biotinylated peptide was indistinguishable from that of the unmodified P^BLV^-391, indicating that potential dimerization of the peptide through inter-molecular disulphide bonding does not influence peptide potency (Figure 4C). The first 3 amino acids of the BLV peptide, which includes the two cysteine residues and an adjacent phenylalanine, are conserved between HTLV-1 and BLV. Given the data obtained for the BLV peptide it is surprising to note that substitution of the cysteines with alanine did not affect the activity of the HTLV-1 inhibitor P^cr^-400 [22]. Thus it seems that, at least for the BLV peptide, the first 3 amino acids aid peptide solubility and contribute in an important but, as yet, ill-defined way to the binding or orientation of the peptide within the target-binding site on TM.

### Two conserved leucines are essential for the inhibitory activity of P^BLV^-391

Leucine residues in P^cr^-400 play a key functional role in peptide activity [20]. The crystal structure of the HTLV-1 TM [16] reveals that within the LHR several leucine and isoleucine residues reach down into deep pockets within the groove of the coiled coil. It appears that the LHR-derived peptide P^cr^-400 makes similar contacts with the coiled coil and that these contacts are necessary for stable binding of the peptide to the coiled coil and thus are critical to the inhibitory activity of the peptide [22]. Intriguingly, some but not all of these leucine and isoleucine residues are conserved between the LHRs of HTLV-1 and BLV. We therefore sought to determine the importance of these conserved residues to the inhibitory properties of the BLV LHR-mimetic peptide. Two peptides were synthesized, P^BLV^-L/A in which all leucines were substituted with alanine, and P^BLV^-L404/410A in which the Leu404 and Leu410 of BLV envelope were replaced by alanine (Table 1) these particular leucines are equivalent to the well-conserved Leu413 and Leu419 of HTLV-1 isolates. Syncytium interference assays revealed that compared to the parental peptide (P^BLV^-391) the alanine-substituted peptides were severely compromised in their ability to inhibit membrane fusion (Figure 4D); in particular, P^BLV^-L/A did not exhibit any discernible inhibition up to 20 μM (Figure 4D) or above (data not shown). Hence, the leucine residues are important to peptide function. Moreover, although P^BLV^-L404/410A was just as soluble as the parental peptide (Table 1), P^BLV^-L404/410A also failed to display any fusion-blocking activity up to 20 μM (Figure 4D); indicating that the leucines equivalent to BLV envelope residues 404 and 410 are particularly important to the inhibitory properties of the LHR-mimetic peptide.

### A model for the BLV trimer-of-hairpins

Our analysis reveals that for the ectodomain of the TM the majority of the amino acid residues that are conserved between HTLV-1 and BLV map to the interacting surfaces of the trimer-of-hairpins. Moreover, a BLV homologue of the HTLV-1 LHR-derived peptide inhibitor also exhibits robust but highly specific inhibitory activity against BLV-induced membrane fusion. Significantly, conserved leucine residues are critical to the inhibitory activity of both peptides. Encouraged by these results and to gain greater insight into the mechanism of fusion and the likely contacts made by P^BLV^-391 with the coiled coil, we constructed a homology model of the BLV trimer-of-hairpins that is based on the crystal structure of the HTLV-1 TM (Figure 1B) [16].

**Figure 5:**
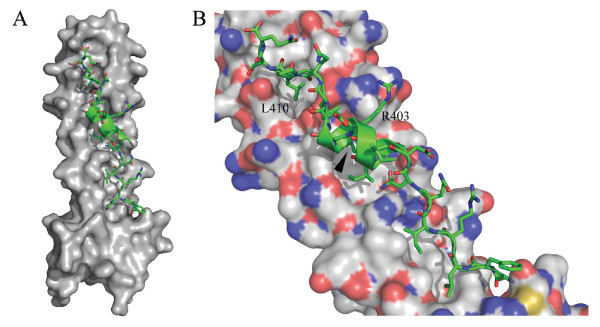
**Homology model of the BLV core coiled-coil and the interacting LHR**. The protein sequence of BLV TM was modelled onto the HTLV-1 TM ectodomain structure (PDB ID 1MG1). (A) The predicted BLV core coiled-coil is shown as a space-filling model in grey with the LHR in green. (B) Detail of the coiled coil in blue, grey and red, with the C-terminal section mimicked by P^BLV^-391 shown as a green ribbon, the predicted position of relevant side chains are shown as sticks. The membrane proximal region is uppermost. The arrowhead marks the position of Leu404.

Having identified the predicted BLV coiled-coil (Figure 1A), the Clustal-W alignment of the TM ectodomain sequences of BLV and HTLV-1 (Figure 1A) permitted the substitution of the BLV residues onto the HTLV-1-derived scaffold, consisting of the complete trimer of N-helices and a single LHR. The geometry of the crude model was improved by simulated annealing and energy minimisation in explicit solvent with the GROMACS (Groningen Machine for Chemical Simulations) package using the GROMOS96 43a1 force field [34]. It should be noted that, compared to the HTLV-1 trimer of hairpins, there are two additional residues in the predicted BLV chain-reversal region at positions 380 and 381 of BLV envelope. Since these residues are within a flexible loop there is insufficient information to model these residues with any degree of accuracy therefore these residues are omitted in the current model. Nonetheless, the restraint provided by the disulphide bond between Cys384 and Cys391 coupled with a high level of sequence conservation within the heptad repeat region and within the LHR suggests that the model is likely to be a reasonably accurate representation of the interaction between the LHR and the coiled coil. The model for the BLV coiled coil and LHR is presented in Figure 5A.

Consistent with the sequence alignment and the structure of the HTLV-1 TM ectodomain (Figure 1), the BLV TM model indicates that Leu394 and Ile396 likely project into a hydrophobic pocket at the membrane-distal end of the core coiled-coil (Figure 5B). It also implies that Ile401, Leu404 and Leu407, which all lie on the same side of the putative α-helix of the LHR, are oriented such that they project into the groove of the coiled coil. Notably, Leu410 is predicted to make a significant contact with a deep pocket situated towards the membrane-proximal end of the core coiled-coil. Therefore, the BLV coiled coil and LHR model is highly consistent with the experimental data and provides a molecular explanation for the loss of activity associated with substitutions in the BLV LHR-derived peptide.

### Substituting an arginine residue for an alanine in P^BLV^-391 results in a more potent peptide inhibitor

**Figure 6:**
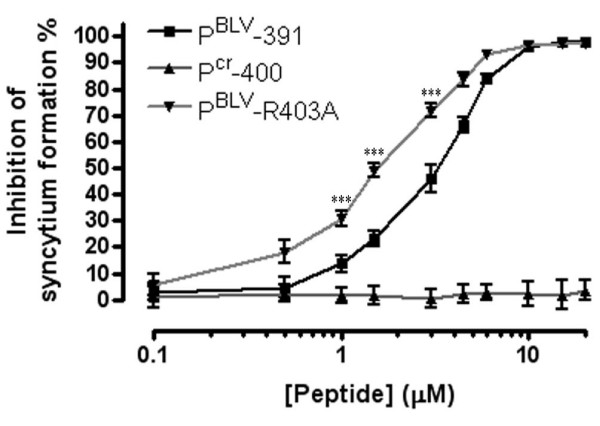
**Substitution of a single arginine residue with alanine yields an improved inhibitor**. The syncytium inhibition activity of the peptides P^cr^-400, P^BLV^-391 and the derivative peptide P^BLV^-R403A was examined. The percentage syncytium inhibition following co-incubation of cells with the peptides is shown. Syncytia were counted in 10 low-power light microscope fields. Data points show the mean ± SD of triplicate assays. The asters show the data points for which the *p *values were calculated (see main text).

The accumulated experimental data correlate well with the structural model, implying that predications based on the BLV trimer-of-hairpins model are likely to be informative. The homology model of the BLV TM ectodomain (Figure 6) suggests that Arg403, a residue within the predicted α-helix of the LHR and mimicked by P^BLV^-391 peptide, may be electrostatically unfavourable for efficient binding of the C-terminal LHR into the groove of the core coiled-coil. We predicted that removing this unfavourable charge interaction would improve the binding of the peptide to the BLV coiled coil and thereby improve the inhibitory activity of the peptide. We therefore synthesized a peptide, P^BLV^-R403A, which incorporated an alanine residue in place of the arginine equivalent to Arg403 of Env (Table 1). As anticipated, substitution of the arginine residue resulted in a modest but highly consistent and significant (*p *< 0.0001, Student's *t*-test) improvement in peptide potency when compared to P^BLV^-391. The peptide P^BLV^-R403A is more than twice as potent as P^BLV^-391 in syncytium interference assays, with a calculated IC_50 _of 1.56 ± 0.05 μM compared to 3.49 μM ± 0.03 μM for P^BLV^-391 (Figure 6). The data show that a single amino-acid substitution in the predicted short α-helix of the LHR-mimetic peptide increases the ability of the peptide to block membrane fusion and provides further support for the utility of the model of the BLV TM core.

## Discussion

Experimental evidence points towards a remarkably conserved mechanism by which virally encoded envelope glycoproteins catalyse membrane fusion and facilitate delivery of the viral core into the target cell [13,14]. The structures of several class 1 fusion proteins reveal a characteristic "trimer-of-hairpins" motif believed to represent a late or post-fusion conformation [16-19,35-37]. Investigating the way in which envelope proteins fold from a rod-like, pre-hairpin intermediate into the trimer-of-hairpins to pull the viral and cellular membranes together is important not only for our understanding of viral entry but also for the development of therapeutically relevant inhibitors of this process.

The protein sequences of the TM ectodomains of BLV and HTLV-1 display a striking level of conservation. By scrutinizing the position of conserved residues in the context of the HTLV-1 six-helix-bundle structure, we have found that the majority of the conserved residues map to the interacting surfaces of the LHR and core coiled-coil. It is interesting to note that there are several non-conserved residues within the LHR of each virus; significantly, these modifications are mirrored by compensating substitutions within the specific area of the core coiled-coil with which the variant residue interacts (Figure 1C) and consequently, the association with the coiled coil is maintained. It appears that in order to support variation and speciation but to maintain biological function complementary regions of the fusion proteins have evolved in parallel. The greatest functional constraint and therefore most highly conserved regions map along the interacting surfaces of the trimer-of-hairpins. Conversely, regions of the TM that are likely exposed to the aqueous environment both during and after fusion exhibit considerable divergence and display relatively few amino acids in common. Such changes may reflect strong selective pressures exerted on the virus, perhaps due to the need for particular regions of the TM to interact functionally with the relatively divergent surface glycoproteins of the respective viruses. Alternatively, the selective pressure may be due to the differing immunological environments of the respective hosts. It is worth noting, that the TM and the trimer-of-hairpins of HTLV-1 are immunogenic [38,39], that antibodies targeting TM often recognise non-neutralizing conformational epitopes [39,40], and that trimer-of-hairpin structures are frequently displayed on the surface of infected cells [40]. Whether or not these features of the TM contribute to the pathogenesis or immune evasion of leukaemia viruses remains to be determined.

The HTLV-1-derived LHR-based peptide is able to inhibit membrane fusion mediated by the divergent envelope of HTLV-2 and, given the level of conservation between the HTLV-1 and BLV TM ectodomain, we anticipated that the HTLV-1-derived peptide P^cr^-400 would also inhibit the fusogenic activity of BLV envelope. Surprisingly, although P^cr^-400 is an extremely effective inhibitor of HTLV-1-mediated fusion, the peptide had no detectable activity in BLV syncytium interference assays. Moreover, the BLV LHR-based peptide P^BLV^-391 does not inhibit HTLV-1 envelope-catalysed syncytium formation. Sequence alignment and homology modelling (Figures 1 and 5) indicate that within the first eight residues only two residues differ between the HTLV-1 and BLV peptides and these residues are likely to be solvent exposed and unable to contribute to the interaction with the core coiled-coil. The residues that determine the specificity of inhibition are therefore located within or overlapping the short α-helix or C-terminal leash segments of the peptide. In terms of peptide function, it is clear that the putative α-helix within the central region of these peptides is important for inhibitory activity. Nonetheless, both the N- and to the C-terminal leash residues contribute to the inhibitory properties of the peptide as deletion of these regions severely attenuates inhibitory activity. The structure of residues C-terminal of Asn421 in the HTLV-1 TM (equivalent to Gln412 of BLV Env) has not been resolved [16]. Consequently, it is not yet possible to account in molecular terms for the conserved interactions beyond this point. However, our data highlight a number of features that play a key role in the biological activity of the BLV-derived peptide. The first three N-terminal amino acid residues appear to be critical to activity. Given the orientation of the phenylalanine residue in the BLV TM model and the equivalent Phe402 in the crystal structure of HTLV-1 TM, it is unlikely that this side chain directly contributes to the interaction with the coiled coil. Consistent with this view, Maerz et al. [41] have demonstrated that Phe402 likely plays a structural role in pre-fusogenic envelope and is required for envelope processing, but likely becomes solvent exposed during assembly of the fusion-associated trimer-of-hairpins structure [41]. Furthermore, although disulphide bonding regulates TM function [11,12] and association with the SU subunit [10], the adjacent cysteines at the N-terminus of P^cr^-400 are not required for disulphide formation, for binding to the coiled-coil, or for inhibitory activity [22]. Similarly, modification of the adjacent cysteine residues in the BLV-derived peptide reveals that disulphide formation is not required for coiled coil binding or inhibition of membrane fusion. The apparent requirement for the cysteine residues for functional activity of the BLV-derived peptide may reflect an intrinsic difference between BLV and HTLV-1 peptide target interactions. Currently, our preferred view is that the N-terminus of the BLV peptide aids alignment of the adjacent peptide sequences relative to the target-binding site on the coiled coil.

A recurring theme in the interaction of the C-terminal helix of the trimer-of-hairpins with the coiled coil of viral fusion proteins is the interaction of non-polar side chains with deep pockets on the coiled coil [16-18,35,36,42]. The model for the BLV trimer-of-hairpins suggests that this is also the case for BLV and this interpretation is supported by the peptide inhibition data. The model suggests that a series of leucine residues, which include L404, L407 and L410, make contact with the coiled coil. Moreover, the inhibitory activity of P^BLV^-391 is completely abrogated following substitution of all the leucine residues with alanine. Similar results have been observed for the P^cr^-400 inhibitor of HTLV-1 [20]. In particular, two leucines, Leu413 and Leu419, are important for the inhibitory activity of P^cr^-400 [22]. Leucine 413 is situated within the short α-helix, whereas Leu419 is situated within the C-terminal leash-like domain. Significantly, both of these leucine residues are conserved in BLV, at positions 404 and 410 respectively, and the model for the BLV trimer-of-hairpins suggests that they are located in areas of similar structure. Importantly, substitution of these residues in P^BLV^-391 results in a non-functional peptide. This is a significantly more dramatic outcome than is observed for specific substitutions at each of these residues in P^cr^-400 [22] and suggests that disruption of both of the potential contacts made with the coiled coil has a profound cumulative effect on loss of peptide activity. Given that these leucines are critical to the inhibitory properties of the LHR-mimetic we suspect, and are currently testing the view, that within envelope such substitutions would severely impair envelope-mediated membrane fusion. Our data also reveal that P^BLV^-391 is significantly less potent against BLV than the comparable peptide (P^cr^-400) against HTLV-1. The structure and model of the HTLV-1 and BLV TM suggests a plausible explanation for this observation in that, relative to the HTLV-derived peptide, the BLV peptide displays a smaller surface area available for interaction with the core coiled-coil. In addition, non-conserved residues within the HTLV-1 peptide may contribute disproportionately to the stability of the interaction between the HTLV-1 peptide and the core coiled-coil. The model and accumulated data also underscore the importance of a deep pocket that is situated towards the membrane-proximal end of the trimer-of-hairpins and is conserved between leukaemia viruses. The peptide inhibitors engage this pocket and this interaction appears to contribute substantially to the stability of peptide association with the coiled coil and is required for optimal inhibitory activity. The data provides further validation of the BLV coiled coil and LHR model and reveals that conserved hydrophobic amino acid side-chains within the helical and non-helical regions mediate interaction of the peptide inhibitors with their target.

An intriguing finding of this study is that, directed by analysis of the model structure, an improved inhibitor of BLV envelope-mediated membrane fusion can be generated by the substitution of a single amino acid residue, Arg403, with alanine. A similar observation has been made for the Ile412 residue of the HTLV-1 fusion inhibitors [22]. Interestingly, the relative location of these beneficial substitutions is conserved: the BLV residue Arg403 and the HTLV-1 residue Ile412 are immediately N-terminal of an important coiled-coil contact mediated by a conserved leucine residue. It is likely that the substitutions relieve a steric and/or electrostatic clash between the peptides and the relevant viral core coiled-coil, and thereby allow the adjacent leucine residue to dock more effectively with the coiled coil. For BLV, the clash between Arg403 and the coiled coil is highlighted in the model of the trimer-of-hairpins (Figure 5B), and this structure is validated by the collected experimental data. Surprisingly, the data derived from the peptide inhibitors identifies a conserved position at which a residue impedes assembly of the trimer-of-hairpins. It appears that during evolution two related but diverging viruses have maintained non-optimal residues within the LHR and that the LHR has not been selected for the best possible fit with the coiled coil. It seems strange not only that such clashes occur, but that they occur in ostensibly the same place. Perhaps, these non-optimal residues act to modulate the fusogenic activity of the TM. It is worth noting that highly fusogenic or readily activated fusion proteins have been described for a number of viruses and these proteins display an array of mutations or deletions, implying that fusogenic activity is modulated by multiple regions of envelope [43-46]. Of course, it is also possible that the non-optimal residues for LHR association with the coiled coil modulate envelope activity at an earlier pre-fusogenic stage of envelope assembly. Studies are currently underway to test these ideas. Importantly, the ability to remove residues that hinder LHR:coiled coil interaction provides an opportunity to design peptides with "super-binding" characteristics and thereby pave the way towards more drug-like HTLV-1 entry inhibitors.

BLV is prevalent among cattle throughout many regions of the world [3]. The combined effect of decreased milk production, mortality due to lymphoma, reduced productive lifespan and increased susceptibility of infected cattle to opportunistic pathogens has significant economic ramifications [3]. Our data indicate that the core coiled-coil of gp30 is exposed at least transiently during the fusion process and is accessible to a small inhibitory peptide and that inhibitory peptides will be of significant utillity in the analysis of BLV entry into cells. Moreover, it will be interesting to determine if the BLV coiled coil is also accessible to neutralising antibodies and whether coiled-coil-based immunogens could be of value as components of a subunit vaccine to prevent BLV transmission between animals. Although retroviral TM displays significant resistance to neutralisation by coiled-coil-specific antibodies [47,40] recent efforts indicate that such hurdles can be successfully overcome [48]. Moreover, attenuated BLV strains provide long-term protection against experimental BLV infection of cattle [49]; and an HTLV-1 envelope-derived subunit vaccine candidate provides significant protection against virus challenge in primate models [50]. The accumulating evidence therefore suggests that a subunit vaccine based on viral envelope may be an achievable objective for prophylactic treatment against leukaemia virus infections.

Our data further define a membrane-proximal region of TM that is conserved between BLV and HTLV-1, which has potential as an anti-HTLV-1 drug target. This study demonstrates that comparative analysis of BLV and HTLV-1 induced membrane fusion will provide significant insight into envelope function and ultimately will be of value in the quest for compounds that block HTLV-1 entry into cells.

## Competing interests

The authors declare that they have no competing interests.

## Authors' contributions

DL performed the experiments and helped to draft the manuscript, AS provided technical expertise in molecular modeling, DvA provided assistance and technical expertise in structural analysis, DWB designed the experiments and wrote the manuscript. All authors read and approved the final manuscript.
